# Systemic Sclerosis and Multiple Cancers of the Female Genital Tract: Prolonged Survival following Current Treatment Strategies

**DOI:** 10.1155/2011/392068

**Published:** 2012-01-04

**Authors:** Georgios Androutsopoulos, Georgios Adonakis, Athanasios Tsamandas, Andreas Andonopoulos, Georgios Decavalas

**Affiliations:** ^1^Department of Obstetrics and Gynecology, Medical School, University of Patras, 26500 Rion, Greece; ^2^Department of Pathology, Medical School, University of Patras, 26500 Rion, Greece; ^3^Division of Rheumatology, Department of Internal Medicine, Medical School, University of Patras, 26500 Rion, Greece

## Abstract

*Background*. Systemic sclerosis is a rare, chronic, multisystem, and autoimmune disease. There is an overall increased risk of malignancy in patients with systemic sclerosis. However, multiple cancers of the female genital tract in patients with SSc are a very rare event. Our aim is to present a case of SSc and multiple cancers of the female genital tract, with prolonged survival following current treatment strategies. *Case*. The patient, a 43-year-old nulliparous premenopausal Greek woman suffering from systemic sclerosis, presented with a history of abdominal pain and abnormal uterine bleeding. She underwent total abdominal hysterectomy with bilateral salpingo-oophorectomy, total omentectomy, appendectomy, and pelvic lymph node dissection. The histopathology revealed synchronous primary cancers of the endometrium and left ovary. The final diagnosis was stage Ib endometrial cancer endometrioid type and stage IIIc ovarian cancer endometrioid type. She underwent postoperative adjuvant chemotherapy and remains well without evidence of disease 89 months after initial surgery. *Conclusion*. Although our patient was diagnosed at advanced stage disease, prolonged survival may be related with radical surgery and postoperative adjuvant chemotherapy according to current treatment strategies.

## 1. Introduction

Systemic sclerosis (SSc) is a rare, chronic, multisystem, and autoimmune disease [1]. It is characterized by humeral and cellular immune dysregulation with excessive collagen and extracellular matrix deposition and structural alterations in microvasculature [1, 2]. It most commonly affects skin and internal organs (lungs, oesophagus and heart) and causes significant morbidity and mortality [1, 2].

There is an overall increased risk of malignancy in patients with SSc [1, 3, 4]. However, multiple cancers of the female genital tract in patients with SSc are a very rare event [1, 3, 5, 6].

Our aim is to present a case of SSc and multiple cancers of the female genital tract, with prolonged survival following current treatment strategies.

## 2. Case Presentation

The patient, a 43-year-old nulliparous premenopausal Greek woman, presented to the Department of Obstetrics and Gynecology of the University of Patras Medical School with a complaint of abdominal pain and abnormal uterine bleeding. Her surgical history was unremarkable. She was suffering from SSc the last 21 years. She received methotrexate, D-penicillamine, and corticosteroids after initial diagnosis of SSc. Her family history revealed no evidence of cancer among the first-degree relatives.

On gynecologic examination, there was a palpable pelvic mass. There were no palpable inguinal lymph nodes, and the rest of pelvic examination was normal.

Preoperative computer tomography (CT) of the abdomen and pelvis and abdominal ultrasound (U/S) revealed an intra-abdominal mass 15 × 15 × 12 cm. Preoperative computer tomography (CT) of the chest, chest X-ray, intravenous pyelography (IVP), colonoscopy, and urethrocystoscopy were normal. Preoperative CA-125 was elevated as 426 U/mL.

On exploratory laparotomy, the left ovary was markedly distended, measuring 20 × 15 × 10 cm. Frozen section showed malignancy, and the patient underwent total abdominal hysterectomy with bilateral salpingo-oophorectomy, total omentectomy, appendectomy, and pelvic lymph node dissection.

**Figure 1:**
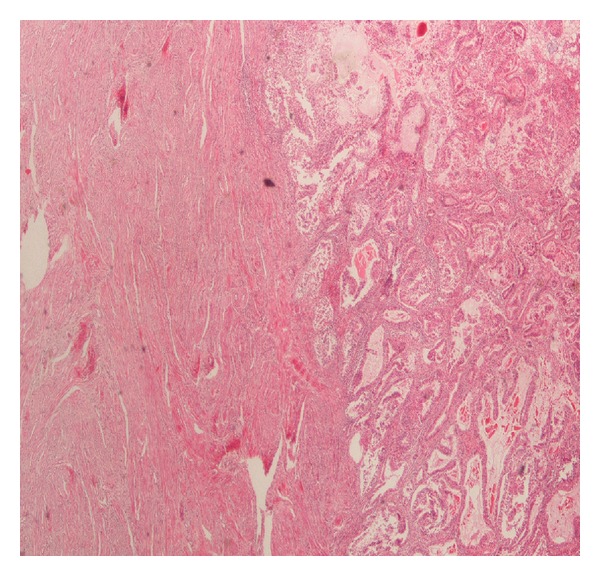
Microphotograph from a section of the endometrial tumor (endometrioid type carcinoma) (H&E ×250).

**Figure 2:**
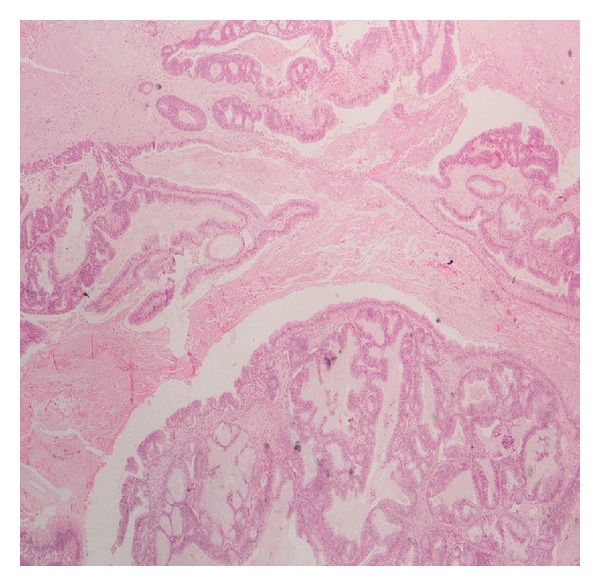
Microphotograph from a section of the ovarian tumor (endometrioid type carcinoma) (H&E ×250).

The histopathology revealed synchronous primary cancers of the endometrium and left ovary. The endometrial tumor invades less than one half of myometrium (Figure 1). The ovarian tumor invades and ruptures the capsule of the left ovary, invades left fallopian tube, and extents to the omentum (Figure 2). The right ovary was normal. The peritoneal washing smear was negative for malignant cells. Tumor cells in both primary cancers were positive for vimentin, cytokeratin, epithelial membrane antigen, estrogen receptor, progesterone receptor, CA-125, CA19-9, and B72.3 and negative for CEA.

The final diagnosis was stage Ib endometrial cancer endometrioid type and stage IIIc ovarian cancer endometrioid type.

The patient underwent postoperative adjuvant chemotherapy. She received six courses of carboplatinum (AUC 5) and paclitaxel (175 mg/m²).

Follow up 89 months after initial surgery, with CT of the chest, abdomen, and pelvis, abdominal U/S, chest X-ray, IVP, colonoscopy, and urethrocystoscopy, revealed no evidence of recurrence.

## 3. Discussion

There is an overall increased risk of malignancy in patients with SSc (SIR = 1.5), compared with the general population [1, 3–5]. The incidence of malignancy in patients with SSc ranges from 3.6% to 10.7% [7]. Among them, the overall risk of malignancy is significantly higher in men (SIR = 2.2) than in women (SIR = 1.3) [5].

All subtypes of SSc (diffuse, limited, and overlap) are associated with an increased risk of malignancy [3]. However, there are differences in risk among them. Patients with diffuse SSc have the highest relative risk of malignancy (SIR = 2.73) [3]. Also, patients with limited or other forms of SSc have similar increased relative risks of malignancy (SIR = 1.85) [3].

The most common malignancy in patients with SSc is lung cancer [1, 3–5]. Other less common malignancies in patients with SSc are non-Hodgkin's lymphoma, leukaemia, breast, esophageal, liver, colon, bladder, and nonmelanoma skin cancer [1, 3–5]. However, multiple cancers of the female genital tract in patients with SSc are a very rare event [1, 3, 5, 6].

The pathogenesis of synchronous primary endometrial and ovarian cancers is unclear. The theory of “secondary Müllerian system” has been proposed to explain the observation of multiple similar cancers of the female genital tract [8, 9]. According to this theory, epithelia of the cervix, uterus, fallopian tubes, ovaries, and peritoneal surfaces simultaneously respond to a carcinogenic stimulus [8, 9]. Shared hormonal receptors (estrogen receptors) may be responsible for the development of multiple primary cancers in predisposed tissue [10, 11].

It is also possible that synchronous presence of endometrial and ovarian cancers is an indicator of an etiologically distinct condition [12]. Perhaps patients with SSc have a more fragile genome, and prior genetic damage may predispose them to develop synchronous primary cancers [3–6, 12–14]. Also, the use of immunosuppressive agents (cyclophosphamide, methotrexate, azathioprine, cyclosporine, and corticosteroids) in patients with SSc may predispose them to develop malignancies [3–5, 14]. Our patient received methotrexate and corticosteroids after initial diagnosis of SSc.

Thus, embryologic, hormonal, or other phenomena may be associated with the development of multiple malignancies arising simultaneously in the female genital tract, in patients with SSc [3–6, 8–16].

Patients with synchronous primary endometrial and ovarian cancers tended to be 10–20 years younger than their counterparts with endometrial or ovarian cancer [17]. The median age at diagnosis is 50 years [18, 19]. They had distinct clinical characteristics including young age, obesity, premenopausal status, and nulliparity [18]. The most common presenting symptoms and signs are abnormal uterine bleeding (46%), abdominal/pelvic pain (17%), and abdominal/pelvic mass (13%) [18]. Our patient was 43-year-old premenopausal and nulliparous woman, and the main presenting symptoms were abdominal pain and abnormal uterine bleeding.

Synchronous primary endometrial and ovarian cancers may have a similar appearance or may be of different histologic types [17, 19]. The distinction between metastatic and synchronous primary cancers is relatively easy, when they have different histologic types. However, the distinction is relatively difficult when they share the same histologic features [20, 21]. According to the empirical criteria described in detail by Scully et al., our patient had synchronous primary endometrial and ovarian cancers [21].

Treatment of choice in early-stage patients with synchronous primary endometrial and ovarian cancers is total abdominal hysterectomy with bilateral salpingo-oophorectomy, total omentectomy, appendectomy, and pelvic lymph node dissection [19]. In advanced stage patients, it required more aggressive management with postoperative adjuvant chemotherapy and/or radiotherapy [22–24]. According to current treatment strategies, our patient underwent radical surgery and postoperative adjuvant chemotherapy, as she had advanced-stage disease.

Prognostic factors for synchronous primary endometrial and ovarian cancers are age, stage of ovarian cancer, grade of endometrial cancer, and adjuvant therapy [25].

Patients with synchronous primary endometrial and ovarian cancers endometrioid type have a better median overall survival compared with nonendometrioid or mixed histologic subtypes [18]. Also, patients with synchronous primary endometrial and ovarian cancers have overall 5-year survival of 85.9% and 10-year survival of 80.3% [19]. Our patient also had synchronous primary endometrial and ovarian cancers endometrioid type. However, 89 months after initial surgery, she was in good condition with no evidence of relapse.

The reason for better median overall survival of patients with synchronous primary endometrial and ovarian cancers is not intuitively obvious [19]. Perhaps favourable clinical outcome may be related with the detection of patients at early-stage and low-grade disease with an indolent growth rate [6, 15, 16, 23]. Usually endometrial cancer produces earlier symptoms, so synchronous ovarian cancer may be detected at an earlier stage [6, 15, 16, 18, 23]. Although our patient was diagnosed at advanced-stage disease, prolonged survival may be related with radical surgery and postoperative adjuvant chemotherapy according to current treatment strategies.
